# The Relationship Between Tramadol Use and Cardio Electrophysiological Balance for Postoperative Pain Treatment in General Surgery Patients

**DOI:** 10.3390/medicina60111731

**Published:** 2024-10-22

**Authors:** Hüseyin Yönder, Kenan Toprak, Mehmet Sait Berhuni, Hasan Elkan, Faik Tatlı, Abdullah Özgönül, Baran Yüksekyayla, Hamza Koyuncu, Mustafa Beğenç Taşcanov, Halil Fedai, Metin Ocak, Yakup Arğa, Ali Uzunköy

**Affiliations:** 1 Department of General Surgery, Faculty of Medicine, Harran University, Şanlıurfa 63300, Turkey; drmsaitberhuni@hotmail.com (M.S.B.); dr_elkan@hotmail.com (H.E.); faiktatli-@hotmail.com (F.T.); drozgonul@yahoo.com (A.Ö.); brn_yuksekyayla@hotmail.com (B.Y.); dr.mhkoyuncu@gmail.com (H.K.); aliuzunkoy@yahoo.com (A.U.); 2 Department of Cardiology, Faculty of Medicine, Harran University, Şanlıurfa 63300, Turkey; kentoprak@hotmail.com (K.T.); drbegenc@gmail.com (M.B.T.); halil.fedai@hotmail.com (H.F.); 3 Department of Emergency Medicine, Faculty of Medicine, Samsun University, Samsun 55080, Turkey; mdmocak@gmail.com; 4 Clinic of Cardiology, Viranşehir State Hospital, Şanlıurfa 63700, Turkey; yakuparga@hotmail.com

**Keywords:** tramadol, iCEBc, iCEB, cardiac arrhythmia, cardio electrophysiological imbalance

## Abstract

*Background and Objective*: This study aimed to investigate the relationship between tramadol use and cardio electrophysiological imbalance (iCEB/iCEBc) in general surgery patients with complaints of acute postoperative pain (APP). *Materials and Methods*: In this prospective cross-sectional study, a total of 218 consecutive patients over the age of 18, who underwent surgical procedures in our clinic (postoperative), were included. For analgesic effect, tramadol was administered with an initial total max dose not exceeding 2 mg/kg. A single max dose (100 mg) was given intravenously, infused in 100 cc of saline over 60 min. In all patients requiring analgesia, electrocardiography (ECG) was performed in the supine position with 12 leads at 25 mm/s and 10 mm/mV, immediately before and after tramadol administration. iCEB was calculated as QT/QRS and iCEBc as QTc/QRS. *Results*: A total of 218 patients were included in this study, with 98 of them being male (45%) and the average age being 46.20 ± 17.19 years. The average tramadol dose for analgesic effect was 98.21 ± 7.62 mg. The QT interval (339.17 ± 36.27 vs. 349.88 ± 30.86, *p* = 0.001), QTc interval (407.07 ± 26.36 vs. 419.64 ± 31.78, *p* < 0.001), QRS duration (80.82 ± 11.39 vs. 78.57 ± 9.80, *p* = 0.018), iCEB (4.26 ± 0.69 vs. 4.52 ± 0.70, *p* < 0.001), and iCEBc (5.14 ± 0.86 vs. 5.42 ± 0.79, *p* = 0.001) values significantly increased compared to the baseline immediately after drug administration. Furthermore, the drug dose was identified as an independent predictor that increased iCEBc (β = 0.201, *p* = 0.003). *Conclusions*: Even at single and therapeutic doses, tramadol increases iCEB and iCEBc. Additionally, the drug dose is an independent predictor of increased iCEBc.

## 1. Introduction

Acute postoperative pain (APP) is a common and significant clinical challenge that affects millions of patients worldwide each year. Despite advances in surgical techniques and pain management protocols, an estimated 70% of postoperative patients experience moderate to severe pain, particularly in the immediate postoperative period [1]. Effective pain management is critical not only for improving patient comfort but also for optimizing surgical outcomes, as poorly managed pain can impede recovery, increase the risk of complications, and prolong hospital stays. While optimal pain control requires a multimodal and multidisciplinary approach that combines pharmacological and non-pharmacological strategies, such a comprehensive approach is not always feasible in clinical practice. In many cases, postoperative pain management falls predominantly to the patient’s surgeon, who often relies on pharmacological interventions [2,3].

Opioid analgesics, including synthetic opioids like tramadol, play a pivotal role in managing moderate to severe postoperative pain. Tramadol is a widely used synthetic opioid with dual mechanisms of action: it binds to μ-opioid receptors to provide analgesia, and it inhibits the reuptake of serotonin and norepinephrine, thereby modulating pain pathways in the central nervous system [4]. This dual mechanism of action makes tramadol a popular choice for managing APP, particularly when patients cannot tolerate stronger opioids. However, tramadol’s pharmacological properties also raise concerns about its safety profile, particularly its potential to induce adverse cardiac effects.

A growing body of evidence suggests that opioid analgesics, including tramadol, can have significant impacts on cardiac electrophysiology. Specifically, opioids have been shown to affect ventricular repolarization, which can lead to a range of cardiac arrhythmias [5,6]. Prolongation of the QT interval, a well-recognized marker of ventricular repolarization, is one such arrhythmogenic effect associated with opioids. Several studies have documented that tramadol use, particularly at high doses or in overdose situations, is associated with QT interval prolongation and an increased risk of arrhythmias [7,8,9,10]. In addition to QT prolongation, tramadol has been linked to other electrocardiographic (ECG) changes, such as QRS widening and heart block, which further underscore its potential cardiac risks [8,9,11].

Recent research has highlighted the importance of more comprehensive ECG markers in predicting arrhythmogenic risk. One such marker is the index of cardiac electrophysiological balance (iCEB), which is calculated as the ratio of the QT interval to the QRS duration (QT/QRS). The corrected version of this index, iCEBc (QTc/QRS), accounts for heart rate variations and has been shown to be a robust predictor of ventricular arrhythmias [12,13,14]. iCEB and iCEBc offer a broader perspective on the balance between ventricular depolarization and repolarization, making them potentially valuable tools for assessing arrhythmia risk in patients treated with drugs that affect cardiac electrophysiology, such as tramadol.

Despite the known effects of tramadol on QT prolongation and cardiac rhythm, there is limited research investigating its impact on iCEB and iCEBc, particularly in the context of postoperative pain management. Given the widespread use of tramadol in surgical patients and the growing awareness of its potential arrhythmogenic effects, it is essential to better understand how tramadol influences these novel ECG parameters.

In this study, we aimed to evaluate the effects of tramadol on iCEB and iCEBc in patients experiencing acute postoperative pain following general surgery. By assessing changes in these ECG markers before and after tramadol administration, we sought to determine whether tramadol use is associated with increased arrhythmia risk, even at therapeutic doses, and whether the dose of tramadol correlates with changes in iCEBc.

## 2. Material and Methods

### 2.1. Study Population

This prospective cross-sectional study included 218 consecutive postoperative patients over the age of 18 who underwent various surgical procedures in our clinic. Patients with known chronic heart disease, those with electrolyte imbalance, those using antiarrhythmic drugs, and those diagnosed with rhythm disturbances or arrhythmias on ECG were excluded from the study. The study was approved by the Harran University Clinical Research Ethics Committee (Date: 15 April 2024, Number: HRÜ/24.04.38), and informed consent was obtained from all patients.

For patients requiring analgesia, 12-lead electrocardiograms were obtained in the supine position at 25 mm/s and 10 mm/mV immediately before and after tramadol administration. iCEB was calculated as QT/QRS and iCEBc as QTc/QRS. Tramadol was administered intravenously at a maximum dose of 2 mg/kg (not exceeding 100 mg). Heart rate, oxygen saturation, and blood pressure were monitored for all patients. A numerical rating scale (NRS) is widely used for patient pain documentation [15]. The NRS was not evaluated in our study because patient compliance is difficult in postoperative patients.

### 2.2. Electrocardiography

For all patients, electrocardiograms were recorded in the supine position with 12 leads at 25 mm/s and 10 mm/mV immediately before and after tramadol administration. QT interval, QTc interval, and QRS duration were assessed (Nihon Kohden, Tokyo, Japan). The QT interval was measured from the beginning of the QRS complex to the end of the T wave, and heart rate correction was made using Bazett’s formula. QRS duration was measured from the beginning of the QRS complex to the end of the T wave in seconds. iCEB was calculated as QT/QRS and iCEBc as QTc/QRS.

### 2.3. Statistical Analysis

Statistical analysis was performed using SPSS 22.0 (SPSS Inc., Chicago, IL, USA). The Kolmogorov–Smirnov test was used to assess the normality of continuous variables. Continuous variables were expressed as mean ± SD, while categorical variables were presented as numbers and percentages. Paired t-tests were used to compare pre- and post-procedure variables. We used the formula delta (%) to show the percentage difference in the change in the basic parameters. Δ(%) = (X_2_ − X_1_)/X_2_ × 100. Linear regression analysis was performed to identify significant predictors of cQT/QRS. A *p*-value of <0.05 was considered statistically significant.

## 3. Results

A total of 218 patients were included in this study, with 98 of them being male (45%) and an average age of 46.20 ± 17.19 years. The demographic characteristics and laboratory values of the patients are presented in Table 1.

The average dose of tramadol for analgesic effect was 98.21 ± 7.62 mg. The pre-procedure and post-procedure ECG characteristics are shown in Table 2.

**Figure 1:**
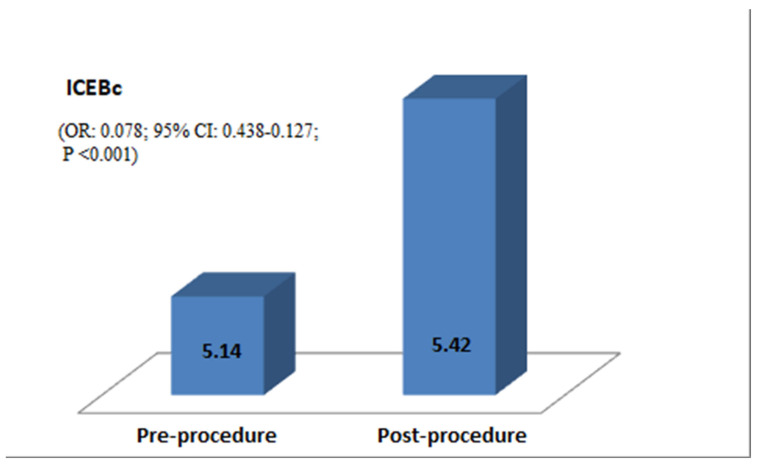
Graph showing the change in iCEBc before and after drug administration.

The QT interval (339.17 ± 36.27 vs. 349.88 ± 30.86, *p* = 0.001), QTc interval (407.07 ± 26.36 vs. 419.64 ± 31.78, *p* < 0.001), QRS duration (80.82 ± 11.39 vs. 78.57 ± 9.80, *p* = 0.018), iCEB (4.26 ± 0.69 vs. 4.52 ± 0.70, *p* < 0.001), and iCEBc (5.14 ± 0.86 vs. 5.42 ± 0.79, *p* < 0.001) significantly increased compared to baseline after stopping drug administration (Figure 1).

Multivariable linear regression analysis was performed to identify predictors of increased iCEBc. The drug dose was identified as an independent predictor of increased iCEBc (β = 0.201, *p* = 0.003) (Table 3).

No significant ventricular or supraventricular arrhythmias were observed during follow-up, and no mortality or morbidity was detected.

## 4. Discussion

The findings of our study provide new insights into the relationship between tramadol use and cardiac electrophysiological balance, particularly in the context of postoperative pain management. We demonstrated that even therapeutic doses of tramadol significantly increase the index of cardiac electrophysiological balance (iCEB) and its corrected version (iCEBc), both of which are strong indicators of arrhythmogenic risk. Additionally, we identified that tramadol dose is an independent predictor of elevated iCEBc values. To the best of our knowledge, this is the first study to directly investigate the effects of tramadol on iCEB and iCEBc in postoperative patients.

Previous studies have extensively documented the effects of tramadol on electrocardiographic parameters, particularly QT and QTc intervals. It is well established that tramadol, like many other opioids, can prolong the QT interval, thereby increasing the risk of ventricular arrhythmias, such as Torsades de Pointes [7,8,9,10]. Our results are consistent with these earlier findings, as we observed a significant prolongation of both the QT and QTc intervals following tramadol administration. However, our study goes beyond simply measuring QT/QTc intervals by incorporating iCEB and iCEBc, newer ECG markers that provide a more comprehensive assessment of the balance between ventricular depolarization and repolarization. These indices are emerging as valuable predictors of arrhythmogenic risk, as they account for the dynamic relationship between ventricular conduction (QRS) and repolarization (QT/QTc) [12,13,16,17]. Our study also contributes to the literature by showing that tramadol has additional electrophysiological effects beyond QT prolongation. Specifically, we observed a significant decrease in QRS duration post-tramadol administration. While QRS duration has been less commonly investigated in the context of opioid-induced cardiac effects, our findings suggest that tramadol may influence ventricular depolarization as well as repolarization. The decrease in QRS duration, combined with QT/QTc prolongation, results in a higher iCEB/iCEBc, which indicates an increased imbalance in cardiac electrophysiology and a potentially higher risk of ventricular arrhythmias.

The observed changes in iCEB and iCEBc have important clinical implications. Both indices are considered robust markers of ventricular arrhythmia risk, with higher values indicating a greater likelihood of arrhythmogenic events [12,13,18]. In our study, we found that tramadol administration led to a statistically significant increase in both iCEB and iCEBc. This suggests that even single, therapeutic doses of tramadol may predispose patients to cardiac dysrhythmias. Furthermore, the fact that tramadol dose was identified as an independent predictor of iCEBc highlights the dose-dependent nature of this risk. These findings are particularly concerning in the postoperative setting, where patients may already be vulnerable to arrhythmias due to surgical stress, electrolyte imbalances, and other perioperative factors. While no overt ventricular or supraventricular arrhythmias were observed in our cohort during the study period, the increase in iCEBc suggests that patients may be at heightened risk for arrhythmias, particularly if higher doses of tramadol are administered or if other arrhythmia-promoting conditions are present. This underscores the importance of careful cardiac monitoring in postoperative patients receiving tramadol, especially in those with pre-existing cardiac conditions or those at higher baseline risk for arrhythmias.

Several mechanisms may explain the observed effects of tramadol on cardiac electrophysiology. Tramadol has dual pharmacological actions: it acts as a μ-opioid receptor agonist and also inhibits the reuptake of serotonin and norepinephrine [6]. Both of these actions are relevant to cardiac function. Increased levels of serotonin and norepinephrine are known to influence cardiac excitability and conduction, potentially leading to arrhythmias [19,20]. Additionally, opioids have been shown to modulate ion channel activity, particularly sodium and potassium channels, which play key roles in the generation and propagation of cardiac action potentials. Nav1.5 flow blockade and increased risk of sudden cardiac death have been reported for some drugs used for noncardiac diseases [21,22]. Recent studies have suggested that tramadol, unlike other opioids, may directly inhibit cardiac sodium channels, particularly the Nav1.5 isoform, which is crucial for ventricular depolarization [23,24,25]. Nav1.5 inhibition can reduce the inward sodium current, leading to QRS prolongation and an increased risk of arrhythmias. Although our study found a decrease in QRS duration, which may seem contradictory, this could be due to the combined effects of sodium channel inhibition and other compensatory electrophysiological changes. For example, tramadol’s effects on serotonin and norepinephrine reuptake may have altered autonomic tone, contributing to the observed changes in QRS duration.

Our findings raise important questions about the safety of tramadol, even when used at standard therapeutic doses, in postoperative patients. While tramadol is generally considered a safer alternative to stronger opioids due to its lower risk of respiratory depression, our data suggest that it may still pose significant risks in terms of cardiac arrhythmias. Clinicians should be mindful of these risks, particularly when prescribing tramadol to patients with pre-existing cardiac conditions or those taking other medications that can prolong the QT interval. Given the widespread use of tramadol in postoperative pain management, further research is needed to confirm and expand upon our findings. Larger, multicenter prospective studies are required to validate the association between tramadol use and increased iCEBc. Moreover, studies examining higher tramadol doses, longer durations of use, and combinations with other medications would be valuable in determining the full scope of tramadol’s cardiac effects. Investigations using continuous cardiac monitoring, such as 24 h Holter recordings, could provide additional insights into the real-time arrhythmogenic potential of tramadol in different patient populations.

### Limitations

Several limitations of our study should be acknowledged. First, this was a single-center, cross-sectional study with a relatively limited sample size, which may limit the generalizability of our findings. Second, we did not perform long-term cardiac monitoring (e.g., 24 h Holter) to capture delayed or transient arrhythmias that might occur after the study period. Third, while we identified tramadol dose as a predictor of increased iCEBc, other potential confounding variables, such as electrolyte disturbances or concurrent use of other medications, may have influenced our results. Comparison with the control group could have strengthened our study. Further research is needed to address these limitations and provide a more comprehensive understanding of the cardiac effects of tramadol.

## 5. Conclusions

In conclusion, our study demonstrates that tramadol, even at therapeutic doses, significantly increases iCEB and iCEBc, both of which are strong predictors of ventricular arrhythmias. Tramadol dose was found to be an independent predictor of iCEBc, suggesting a dose-dependent relationship between tramadol use and arrhythmogenic risk. While no major arrhythmias were observed in our patient cohort, these findings underscore the need for careful cardiac monitoring in postoperative patients receiving tramadol, particularly those at higher risk for cardiac complications. Future research should focus on larger, prospective studies to further investigate the cardiac safety profile of tramadol and its impact on novel ECG markers such as iCEBc.

## Figures and Tables

**Table 1:** Demographic data and laboratory findings of the patients participating in the study.

Variables	(*n* = 218)
Age, years	46.20 ± 17.19
Gender female/male (%)	120 (55)/98 (45)
Body mass index (kg/m^2^)	28.56 ± 6.53
Geographic information	Şanlıurfa/Türkiye
Operation time (minute)	60 (45–100)
ASA score	1.90 ± 0.80
Length of stay (day)	2 (2–4)
Tramadol dose (mg)	98.21 ± 7.62
Glucose (mg/dl)	116.02 ± 41.70
Urea (mg/dl)	28 (21–36)
Creatinine (mg/dl)	0.77 ± 0.20
AST (U/L)	24 (18–30)
ALT (U/L)	22 (16–29)
Sodium (mmol/L)	138.62 ± 3.16
Potassium (mmol/L)	4.09 ± 0.45
Calcium (mg/dl)	8.90 ± 0.93
CRP (mg/L)	1 (0–3)
WBC (×10^3^/µL)	7 (6–9)
Hemoglobin (g/dL)	12.86 ± 2.31
Hematocrit (%)	39.92 ± 6.39

AST: aspartate aminotransferase, ALT: alanin aminotransferase, CRP: c-reactive protein, WBC: white blood count.

**Table 2:** Pre-procedure and post-procedure ECG findings of the patients participating in the study.

Parameters	Pre-Procedure	Post-Procedure	Δ (%)	*p*
Heart Rate (b/pmin)	77.61 ± 15.40	77.91 ± 14.61	0.5	0.505
QT (ms)	339.17 ± 36.27	349.88 ± 30.86	2.41	0.001
QTc (ms)	407.07 ± 26.36	419.64 ± 31.78	2.41	<0.001
QRS (ms)	80.82 ± 11.39	78.57 ± 9.80	−4.5	0.018
ICEB	4.26 ± 0.69	4.52 ± 0.70	3.6	<0.001
ICEBc	5.14 ± 0.86	5.42 ± 0.79	3.3	<0.001

Δ (%): percentage difference.

**Table 3:** Multivariable linear regression analysis representing the significant predictors of ICEBc.

Variables	B	SE	β	t	*p*
Age	0.004	0.003	0.090	1.342	0.181
Body mass index	0.009	0.008	0.073	1.086	0.279
Operation time	0.001	0.001	0.030	0.417	0.667
Hemoglobin	0.027	0.024	0.079	1.150	0.252
Potassium	0.213	0.117	0.116	1.826	0.069
Sodium	0.018	0.017	0.071	1.063	0.289
Tramadol dose	0.021	0.007	0.201	3.036	0.003

## Data Availability

Raw data that support the findings of this study are available from the corresponding author, upon reasonable request.
